# Acceptability of human papillomavirus self-sampling among women living with HIV in sub-Saharan Africa: A systematic review and meta-analysis

**DOI:** 10.1371/journal.pgph.0004605

**Published:** 2025-05-14

**Authors:** Olunike Rebecca Abodunrin, Folahanmi Tomiwa Akinsolu, Oluwabukola Mary Ola, Mobolaji Timothy Olagunju, Vivienne Ferife, Akim Tafadzwa Lukwa, Ishak Kayode Lawal, George Uchenna Eleje, Oliver Chukwujekwu Ezechi

**Affiliations:** 1 Center for Reproduction and Population Health Studies, Nigerian Institute of Medical Research, Lagos, Nigeria; 2 Department of Biostatistics and Epidemiology, Nanjing Medical University, Nanjing, China; 3 Department of Public Health, Faculty of Basic Medical and Health Sciences, Lead City University, Ibadan, Oyo, Nigeria; 4 Chesterfield Royal Hospital, Chesterfield, United Kingdom; 5 Health Economics Unit, School of Public Health and Family Medicine, Faculty of Health Sciences, University of Cape Town, South Africa,; 6 Department of Obstetrics and Gynaecology, Federal Medical Centre, Birnin-Kebbi, Kebbi, Nigeria; 7 Department of Obstetrics and Gynaecology, Nnamdi Azikiwe University Teaching Hospital Nnewi, Awka, Nigeria; 8 Effective Care Research Unit, Department of Obstetrics and Gynaecology, Nnamdi Azikiwe University, Awka, Nigeria; Purdue University, UNITED STATES OF AMERICA

## Abstract

HPV self-sampling has the potential to improve early detection of cervical cancer among women living with HIV (WLHIV), but its acceptability varies, creating implementation challenges, especially in sub-Saharan Africa. This study aims to assess the acceptability of HPV self-sampling among WLHIV. We searched PubMed, Web of Science, CINAHL, Academic Medical Ultimate, Cochrane databases, and Google Scholar. The review protocol was registered with PROSPERO (CRD42022299781). Inclusion criteria were based on population, intervention, comparison, and outcome. Statistical analysis was done with R Studio version 4.3.2, and data abstraction was performed in Microsoft Excel. The analysis included 14 studies on the acceptability of HPV self-sampling among WLHIV. The overall acceptability rate was 73%. The pooled data showed that 94% felt comfortable with self-sampling, 72% found it easy to use, 10% reported pain, 14% felt embarrassed, and 41% felt confident about the process. The study found that a majority of WLHIV accepted HPV self-sampling, a higher rate than in the general female population. Many participants had concerns about the method’s efficacy. This indicates that while WLHIV generally views self-sampling positively, additional education and support are needed to improve their confidence in its accuracy and reliability.

## Introduction

Globally, cervical cancer is the fourth most common cause of female cancer death, with approximately half a million new cases and one-third of a million deaths annually [1–3]. The burden of cervical cancer is disproportionately high in low- and middle-income countries (LMICs), accounting for nearly 90% of cervical cancer-related deaths worldwide [4]. In Africa, cervical cancer is either the leading or second leading cause of female cancer deaths [5]. Despite being one of the few cancers that are 100% preventable and treatable if detected early, about 200,000 new cases and 80,000 deaths occur annually in sub-Saharan Africa (SSA) [5].

With approximately 660,000 new cases and around 350,000 deaths reported in 2022 for cervical cancer among women globally [6], a significant proportion of these cases are directly attributable to high-risk human papillomavirus (HPV) infections [7,8]. HPV 16 and 18 are responsible for most cervical cancers and other HPV-related malignancies in the anogenital and oropharyngeal regions [7–11].

The burden of cervical cancer accounts for 3.3% of all cancer-related deaths worldwide; a significant proportion of these deaths, precisely 24.55%, are concentrated in SSA [11,12]. According to the World Health Organization (WHO), SSA has the highest prevalence of cervical HPV among women, with approximately 24% of women infected [13]. This epidemiological disparity underscores the urgent need for effective and scalable prevention strategies in SSA, where access to routine cervical cancer screening and healthcare infrastructure is often limited [1,2].

Women living with HIV (WLHIV) are at a particularly high risk of developing cervical cancer [14]. Despite advancements in HIV treatment, which have significantly reduced the incidence of other HIV-related cancers, WLHIV remains highly vulnerable to persistent HPV infections and progression to cervical cancer. Studies consistently demonstrate that the prevalence of high-risk HPV and the incidence of invasive cervical cancer are significantly higher among WLHIV compared to their HIV-negative counterparts [15–17]. Ezechi et al. (2023) reported a disproportionately high prevalence of high-risk HPV among WLHIV in SSA, further emphasizing the need for targeted interventions to address this vulnerable population [18].

In SSA, where cervical cancer incidence and mortality rates are disproportionately high [19], the endorsement of HPV self-sampling by WHO holds particular significance [20]. Compared to traditional sampling methods, such as clinician-based sampling, HPV self-sampling offers a promising solution to address the challenges faced in cervical cancer prevention in this region [21]. HPV self-sampling has the potential to improve early detection and reduce the burden of cervical cancer among WLHIV [22].

Despite the potential, the acceptability of HPV self-sampling among WLHIV remains uncertain, particularly in SSA, where social, cultural, and healthcare barriers often complicate the adoption of novel screening methods. Understanding the factors influencing the acceptance of HPV self-sampling is crucial for designing effective interventions and policies to enhance cervical cancer prevention in this high-risk population.

This study aims to systematically review the acceptability of HPV self-sampling among WLHIV and identify factors influencing its uptake. The findings will provide critical insights to inform policies and strategies for improving cervical cancer screening coverage and outcomes, particularly in SSA, where the burden of the disease remains alarmingly high.

## Methods

### Study registration

The study was reported following the Preferred Reporting Item for Systematic Reviews and Meta-analyses (PRISMA) guideline [23]. A 27-item PRISMA checklist is available as an additional file to this protocol in the S1 File. Our protocol was registered in the International Prospective Register of Systematic Reviews (PROSPERO): CRD42024563715.

### Search strategy

PubMed, Web of Science, CINALH, Academic Medical Ultimate, Cochrane, and Google Scholar were systematically searched from January 2000 to January 2024. This timeframe was chosen because the concept of self-sampling for HPV screening has gained significant prominence and undergone substantial development only in the past two decades. A detailed search strategy is outlined in the S2 File.

### Study outcomes

This study examines the acceptability of HPV self-sampling among WLHIV in SSA.

#### Primary outcome.

The proportion of WLHIV who found HPV self-sampling acceptable, as reported in the included studies.

#### Secondary outcomes.

1. Comfort with self-sampling: Participants’ level of comfort while performing HPV self-sampling.
2. Ease of use: Whether participants found the self-sampling process easy to perform.
3. Reported pain: Proportion of participants who reported experiencing pain during HPV self-sampling.
4. Embarrassment: Levels of embarrassment felt by participants when performing self-sampling.
5. Confidence: Participants’ confidence in the accuracy and reliability of the HPV self-sampling method.
6. Agreement between self-collected and clinician-collected samples: Proportion of studies reporting concordance, agreement rate, or diagnostic accuracy between self-collected samples and clinician-collected samples.

### Eligibility criteria

The initial search included all human studies that assessed HPV self-sampling published in English. Studies that specifically targeted WLHIV as participants were considered for the final analysis. However, eligibility for inclusion in this review was limited to studies that reported quantitative data on the acceptance rates of HPV self-sampling. Table 1 presents the study PICOS framework in detail.

**Table 1:** Study PICOS framework.

Component	Description
Population	WLHIV in SSA
Intervention	HPV self-sampling for cervical cancer screening
Comparison	Clinician-based sampling was used as a comparison method.
Outcomes	Primary Outcome: Acceptability of HPV self-sampling among WLHIV in SSA Secondary Outcomes: 1.The factors influencing HPV self-sampling acceptability (Comfort, ease of use, pain, embarrassment, confidence) 2.The agreement between self-collected and clinician-collected samples primarily addresses the reliability and accuracy of self-sampling rather than its acceptability.
Study design	Cross-sectional studies, with data collected from various countries across SSA

### Study selection

The database search results were retrieved and uploaded into Rayyan, an online tool for systematic reviews known for its effectiveness in simplifying the review procedure and facilitating cooperation among reviewers [24].

Following the articles’ deduplication, two authors independently reviewed the titles and abstracts of each study to determine whether they fulfilled the inclusion criteria. Studies that did not meet these criteria were excluded. Subsequently, the full texts of the selected studies were retrieved and scrutinized based on the criteria. The third author resolved disagreements. Details of the study excluded, and their reasons are presented in the S3 File.

### Data extraction

Two authors independently extracted relevant data from the included studies, which a third author then cross-checked to ensure consistency. A standardized data extraction template was developed using a Microsoft Excel worksheet. To assess the reliability of the data extraction process, a pilot data extraction was conducted among the two reviewers on a subset of five included studies, yielding a Kappa value of 0.84, indicating substantial agreement between reviewers, and this measure of agreement highlights the reliability of the reviewers. Following this, data extraction was performed using the pretested template to maintain consistency. The extracted data included the author’s name, year of article publication, title, study design, study location, sample size, and mean age. The rate for acceptability and factors reported in each study were also extracted. Details of the extraction process are presented in the S4 File.

### Statistical analysis

Statistical analysis was conducted using R Studio version 4.3.2, employing the “meta”, “metafor”, “loo”, and “ggplot2” packages for meta-analysis calculations. Specifically, proportions were pooled using the random effects model (REM). Data abstraction was performed using Microsoft Excel. REM was chosen over the fixed-effects model due to the anticipated heterogeneities based on the variations from study to study due to participant age and sociodemographic differences [25]. REM effectively accounts for both within-study and between-study variability. REM assumes that the true effect size may differ across studies. This characteristic makes REM more appropriate for analyses involving heterogeneous data. Subgroup analysis was conducted based on the countries where the studies were conducted.

Summary statistics were expressed as proportion along with 95% confidence intervals (C.I.s). To assess statistical heterogeneity among effect sizes, we conducted Cochran’s Q test, supplemented by the I² statistic. While Cochran’s Q test indicates the presence of heterogeneity through a significant p-value, its sensitivity to the number of studies can lead to either low power with few studies or excessive sensitivity with numerous studies. To address this limitation, we used the I² statistic, which provides a more robust measure of heterogeneity, allowing us to quantify the proportion of total variance attributable to between-study heterogeneity [26]. The statistical significance threshold was set at P < 0.05. We employed funnel plot analysis to visually assess publication bias or small-study effects. In the absence of publication bias, a funnel plot is expected to exhibit a symmetrical, inverted funnel shape, with most of the studies clustering around the overall effect size estimate while smaller studies are scattered more widely. Any asymmetrical distributions or noticeable gaps within the plot may suggest the presence of publication bias.

Sensitivity analysis was also performed using:

1. Baujat plot and External studentized residual (ESR) to detect the outliers.
2. Leave one out analysis to determine the influence of individual studies on the overall effect size estimate.
3. Leave one out diagnostic influential analysis to further identify the potential influential studies suggested from the Baujat plot and ESR.

### Quality of the study assessment

The quality assessment used the appraisal tool for cross-sectional studies (AXIS) [27]. Each study underwent assessment across 20 questions. The AXIS quality assessment tool does not use a numerical rating system for evaluating studies but offers flexibility for users to make an overall quality judgment. However, in this study, the authors categorized papers into three groups according to their overall quality score: 1) scores below 10 indicated low quality, 2) scores from 11 to 15 indicated moderate quality, and 3) scores from 16 to 20 indicated high quality. Although, the AXIS’s criteria may not capture all aspects of study quality, and some items may be subject to interpretation.

Furthermore, the assessment process relies on reviewer judgment, which can introduce subjective variability. To mitigate this, two independent reviewers, trained and calibrated to ensure consistency in applying the AXIS tool, conducted this evaluation. The training process involved a comprehensive review of the AXIS tool’s criteria and guidelines, followed by a pilot exercise where both reviewers independently evaluated a subset of studies. Discrepancies were discussed and resolved by the third reviewer. The outcomes of this assessment are detailed in the S5 File.

### GRADE assessment

The GRADE (Grading of Recommendations Assessment, Development, and Evaluation) approach was used to evaluate the quality of evidence.

1. Participant’s acceptance of self-sampling for HPV testing and
2. Agreement between self-collected and clinician-collected samples for HPV detection across diverse low-resource settings the agreement.

The methodology adhered to standardized GRADE protocols to systematically assess evidence across multiple domains and assign an overall certainty rating to the findings [28,29].

Each study was evaluated against the following GRADE domains:

1. Risk of Bias: Assessed based on the robustness of study design, self-reported data, and potential selection bias.
2. Inconsistency: Evaluated based on variations in reported confidence levels across studies.
3. Indirectness: Determined by the applicability of study findings to the broader population or specific subgroups.
4. Imprecision: Assessed by the adequacy of sample sizes and confidence intervals for confidence measures.
5. Publication Bias: Evaluated by examining the journal’s reputation, transparency in reporting, and evidence of selective reporting.

The outcomes of this assessment are detailed in the S6 File.

## Results

### Selection of studies

**Fig 1:**
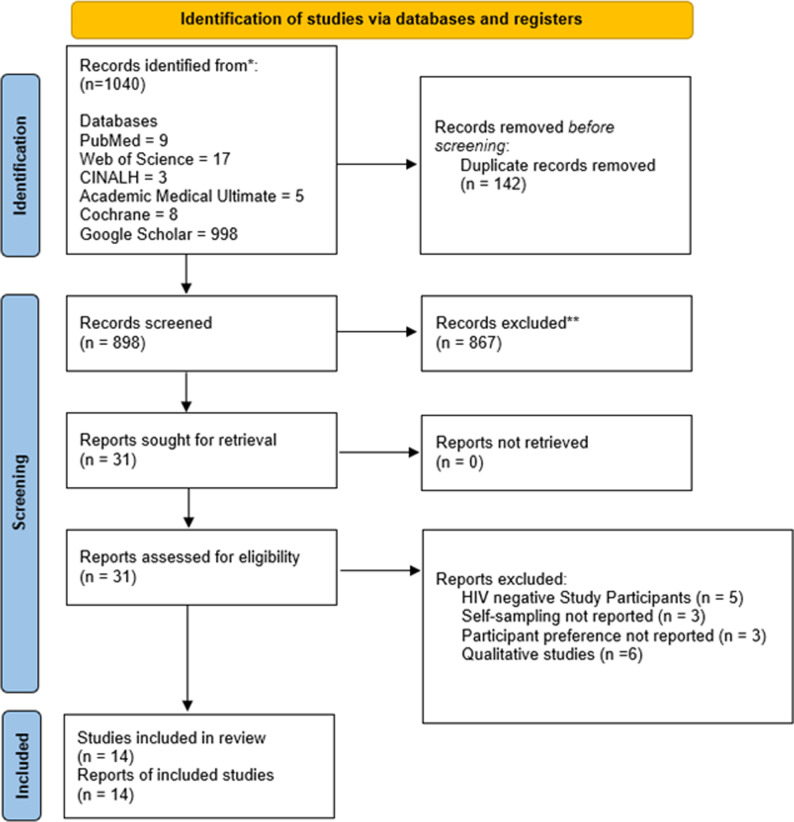
PRISMA flowchart.

1040 articles were retrieved from five databases (PubMed, Web of Science, CINALH, Academic Medical Ultimate, Cochrane) and Google Scholar. After removing duplicates, 898 records were screened for titles and abstracts, of which 867 studies were excluded based on the title and abstract screening. Finally, after thoroughly reviewing the full-text records of 31 studies, 14 met the inclusion criteria (See Fig 1) [30–43].

### Characteristics of included studies

The data extracted are summarized in Table 2. The total sample size of participants across the included studies is 6,649 WLHIV. These studies encompass seven African countries. Most of the studies were conducted in South Africa [24,31,33,36], followed by Kenya [25,26,28]. All the included studies were cross-sectional studies. The mean age of participants across the studies ranged from 18 to 69.

**Table 2:** Characteristics of included studies.

S/N	AUTHORS (YEAR)	COUNTRY	SAMPLE SIZE	MEAN AGE (YEARS)	STUDY TYPE	OVERALL SCORE
1.	Adamson *et al.*, (2015) [30]	South Africa	325	41.6	Cross-sectional study	17
2.	Rositch *et al.*, (2012) [31]	Kenya	286	29	Cross-sectional study	16
3.	Grabert *et al.*,(2022) [32]	Kenya	93	39	Cross-sectional study	14
4.	Kohler *et al.*, (2019) [33]	Botswana	104	45	Cross-sectional study	16
5.	Islam *et al.*, (2020) [34]	Kenya	400	39	Cross-sectional study	15
6.	Sormani *et al.*, (2021) [35]	Cameroon	75	40.6	Cross-sectional study	16
7.	Bansil *et al.*, (2014) [36]	Uganda	3,863	NA	Cross-sectional study	15
8.	Taku *et al.*, (2020) [37]	South Africa	413	46	Cross-sectional study	14
9.	Joseph *et al.*, (2021) [38]	Zimbabwe	280	Range 30–49	Cross-sectional study	15
10.	Mahomed *et al.*, (2014) [39]	South Africa	106	40	Cross-sectional study	18
11.	Obiri-Yeboah *et al.*, (2017) [40]	Ghana	96	44.1	Cross-sectional study	16
12.	Mitchell *et al.*, (2017) [41]	Uganda	91	Range 30–69	Cross-sectional study	16
13.	Mbatha *et al.*, (2017) [42]	South Africa	91	18	Cross-sectional study	16
14.	Nyabigambo *et al.*, (2022) [43]	Uganda	426	Range 30–45	Cross-sectional study	15

### Quality assessment of included studies

According to the appraisal tool, AXIS, the findings indicate that all included studies had well-defined study objectives focused on evaluating the acceptability of HPV self-sampling. Each study employed an appropriate cross-sectional survey methodology aligned with their objectives. However, only two studies [24,37] provided a justified estimation of sample size using statistical methods. The target population was specified, and the sample frame and selection process were deemed suitable across all included studies. In terms of the results, all studies presented sufficient primary data and detailed results for the methods described. Additionally, all studies addressed limitations, conflicts of interest, ethical reviews, and confirmed ethical approval or participant consent (See S2 File).

According to the quality scoring set by this study’s author, twelve out of the fourteen studies [24,25,27–30,32–37] were rated as high quality, while the remaining two studies [26,31] were classified as moderate quality (See Table 2).

### Proportion of HPV self-sampling

The pooled random-effects analysis for the included studies reveals that the acceptance rate of HPV self-sampling for screening among WLHIV is 62% (95% CI 50–73). In addition, the result revealed significant heterogeneity with an I^2^ value of 98% (p < 0.01), indicating substantial variability across the studies.

**Fig 2:**
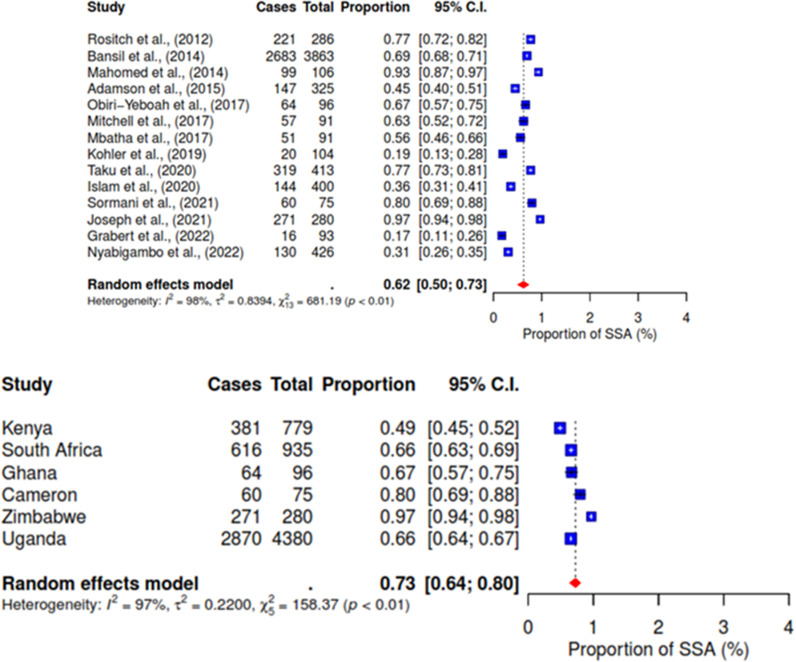
Proportion of HPV self-sampling acceptability and across countries.

The proportion of HPV self-sampling acceptability across countries was 73% (95% CI 64–80) with a significant heterogeneity (I^2^ = 97%; p < 0.01). Specifically, the acceptability rate was high in Zimbabwe 97% (95% CI 64–67), followed by Cameron 80% (95%CI 69–88). However, Kenya’s acceptability rate was low at 49% (95% CI 45–52) (See Fig 2).

### Agreement between HPV self-sampling and clinician sampling

**Fig 3:**
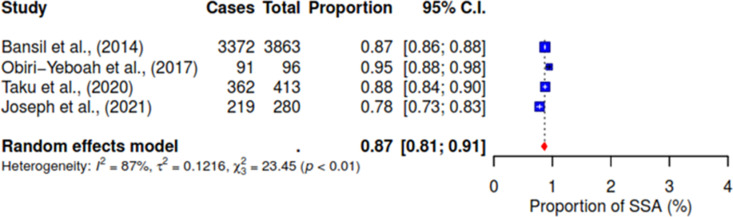
Agreement between HPV self-sampling and clinician sampling results.

Meta-analysis was conducted for the four studies that reported agreement between self-sampling and clinician sampling result. The pooled random effects model shows high agreement between the two methods, 87% (95 CI 81–91; I^2^ = 87%) (See Fig 3).

### Associated factors influencing HPV self-sampling acceptance

**Fig 4:**
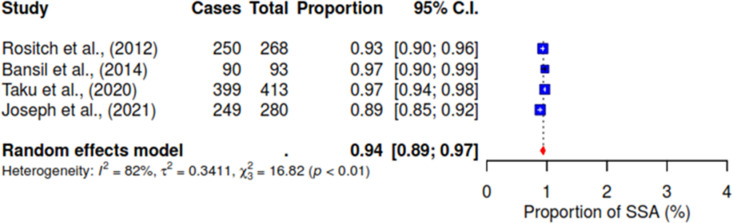
Comfort.

**Fig 5:**
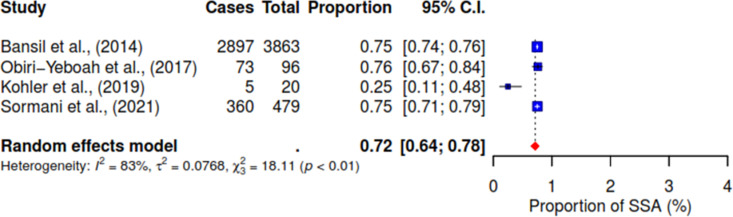
Easy.

**Fig 6:**
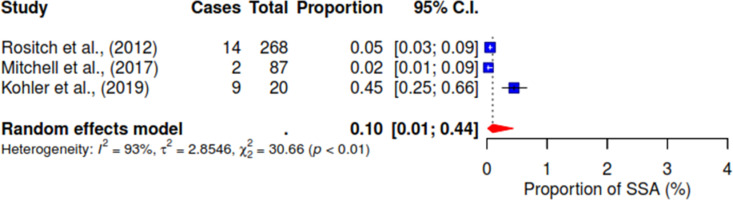
Pain.

**Fig 7:**
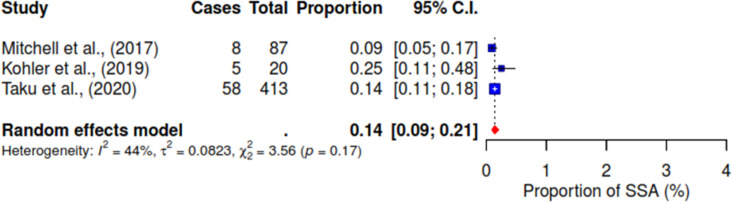
Embarrassed.

**Fig 8:**
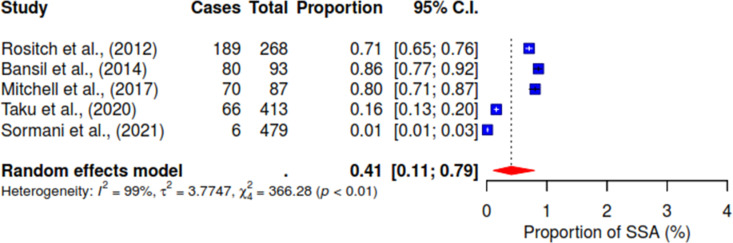
Confident.

Various associated factors reported across the included studies were investigated concerning the acceptance of HPV self-sampling among WLHIV. Notably, from the pooled result, 94% (95% CI 89–97; I^2^ = 82%) of WLHIV expressed comfort with self-sampling for HPV (See Fig 4). Fig 5 shows that 72% (95% CI 64–78; I^2^ = 83%) of WLHIV perceived the self-sampling process as easy to use. In addition, 10% (95% CI 1–44; I^2^ = 93%) reported feeling pain (See Fig 6), and 14% (95% CI 9–21) felt embarrassed during the procedure with insignificant heterogeneity; I^2^ = 41% (See Fig 7). In Fig 8, only 41% (95% CI 11–79, I^2^ = 99%) reported feeling confident about HPV self-sampling.

### Sensitivity analysis to detect outliers and influential studies

#### Proportion of HPV self-sampling acceptability.

**Fig 9:**
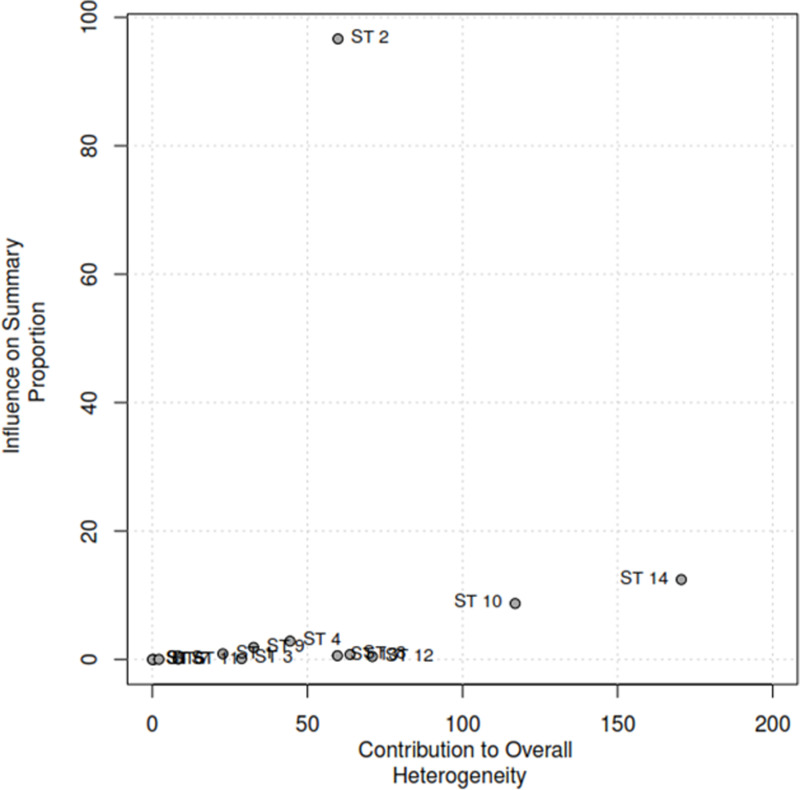
Baujat plot.

**Fig 10:**
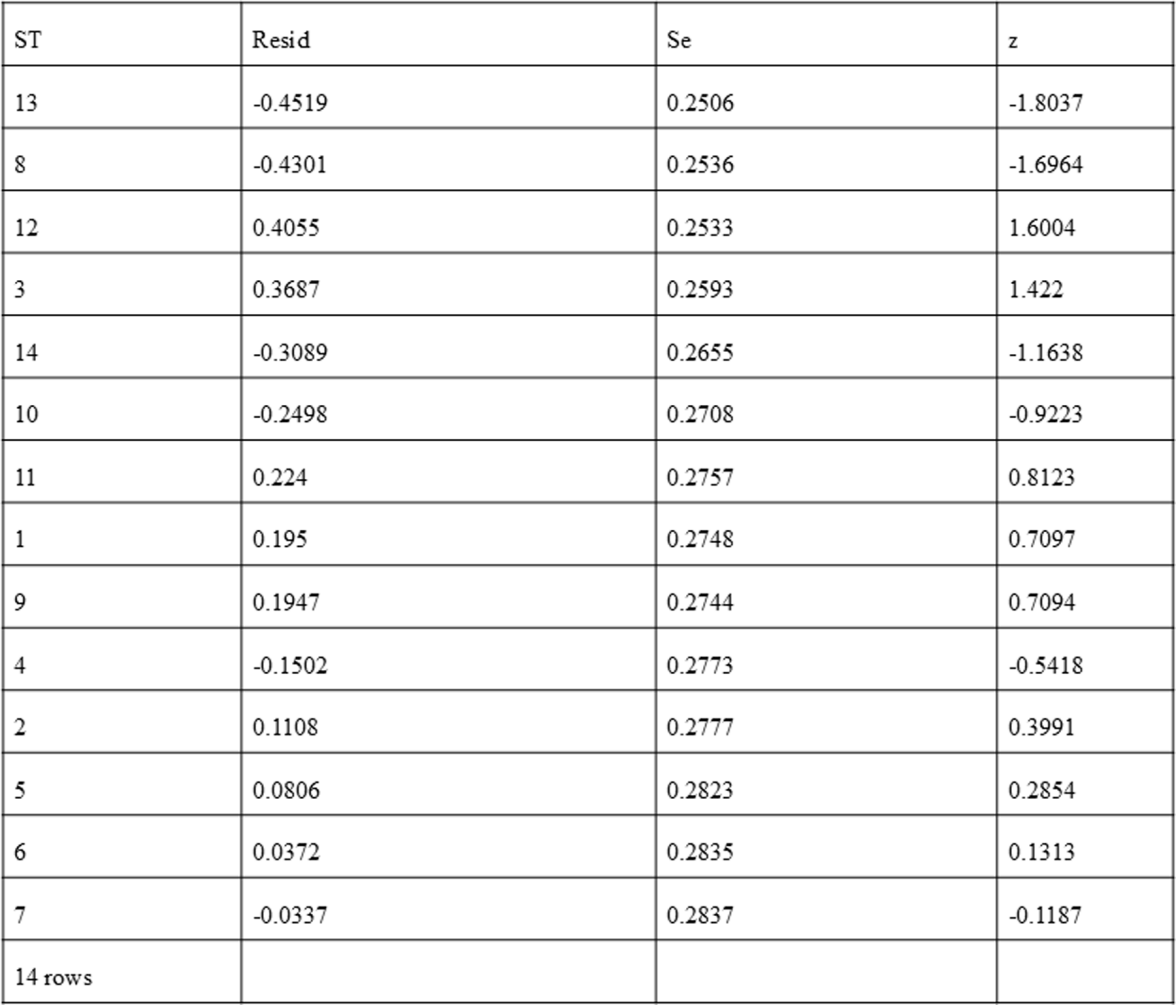
Externally studentized residuals results.

**Fig 11:**
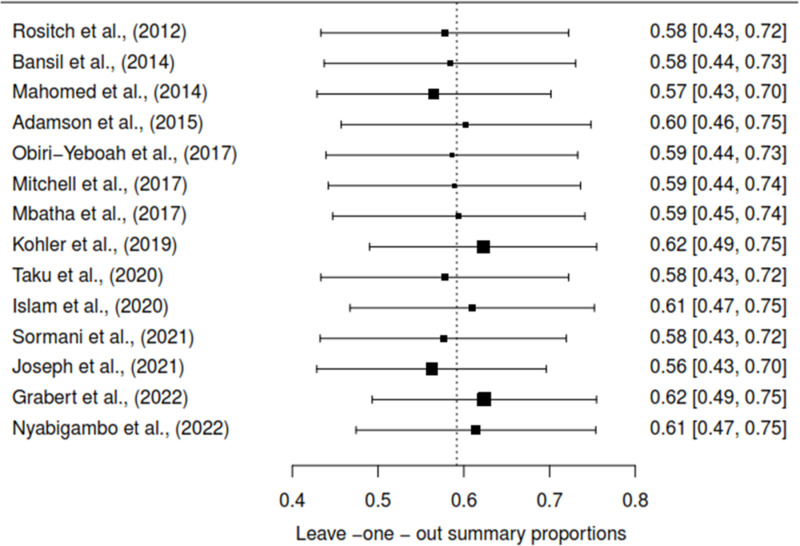
Leave-one-out forest plot.

**Fig 12:**
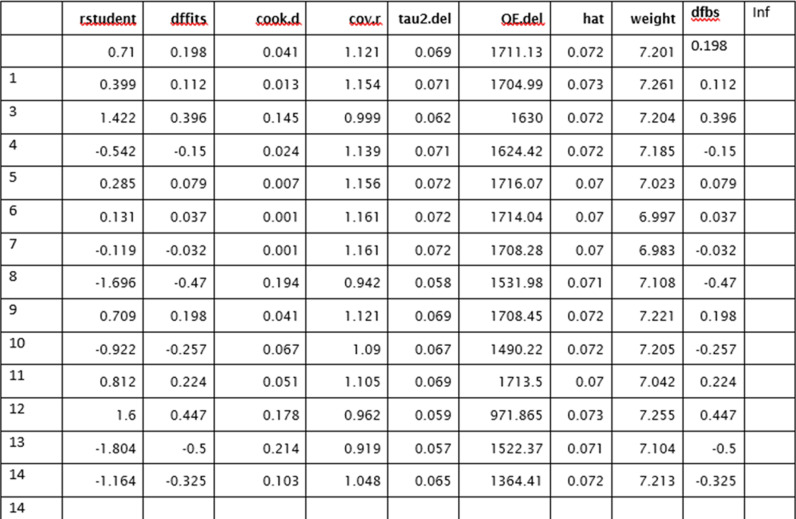
Result of the influential study test.

**Fig 13:**
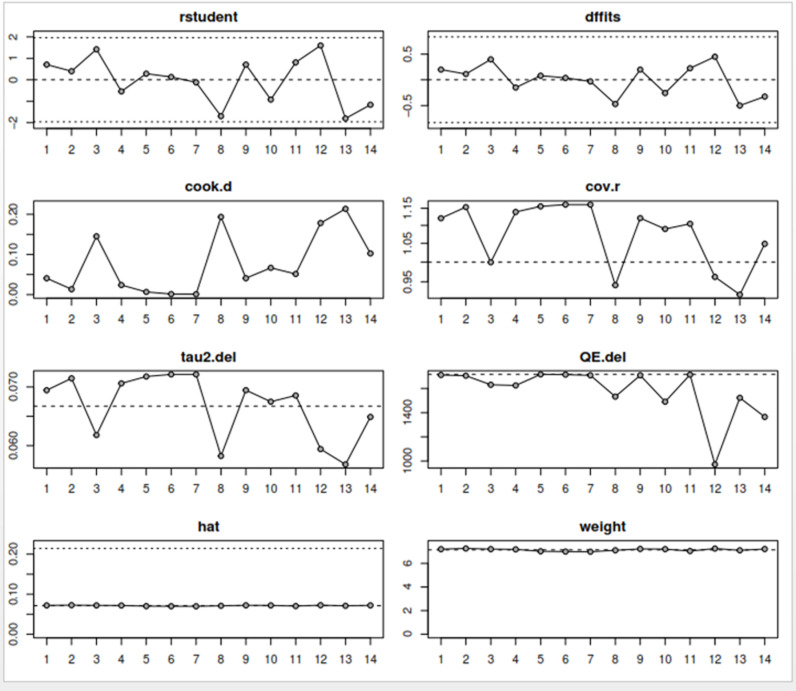
Influential study diagnostics.

The Baujat plot depicted in Fig 9 highlights six studies at the extreme values (>50 contributions to overall heterogeneity) [26–28,30,32,37], which are potentially considered contributing to the overall heterogeneity. Conversely, Bansil et al., (2014) emerge as a notable outlier with a substantial influence on the combined proportion [36] (See Fig 10). However, externally studentized residuals (ESR) result shows no outlier among the included studies (See Fig 10). Moreover, conducting a leave-one-out analysis to assess the impact of individual studies on the overall effect size revealed that excluding studies [26–28,30,32,37] had a shallow effect on the original summary proportion compared to the latter study (See Fig 11). The leave-one-out diagnostics influential analysis indicated that while the suspected studies [26–28,30,32,37] could be classified as outliers, they did not significantly influence the results (See Figs 12 and 13).

#### Proportion of HPV self-sampling across countries.

**Fig 14:**
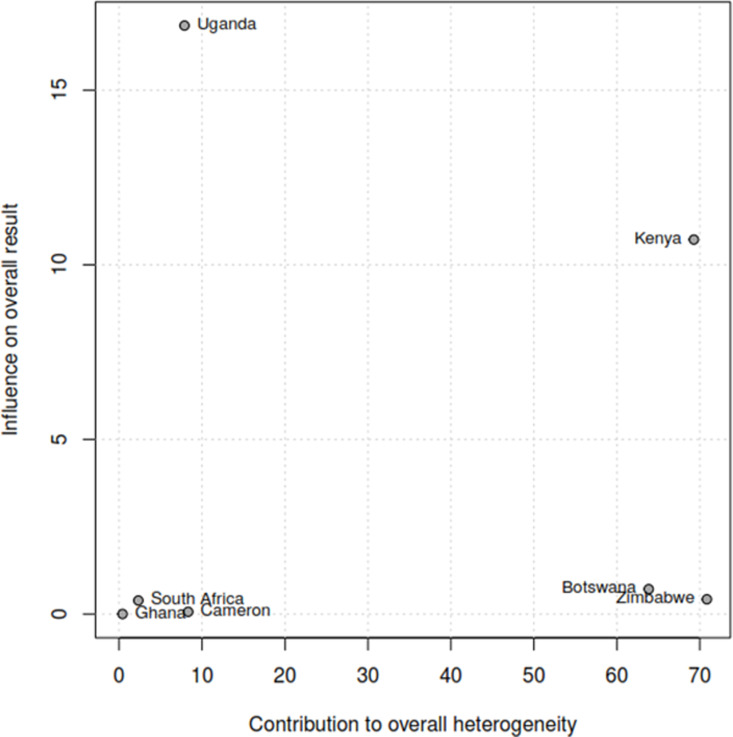
Baujat plot by countries.

**Fig 15:**
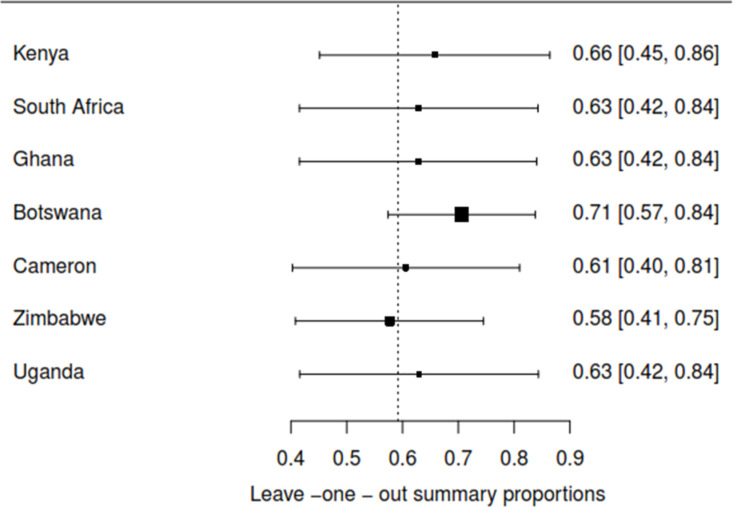
Leave-one-out forest plot by countries.

**Fig 16:**
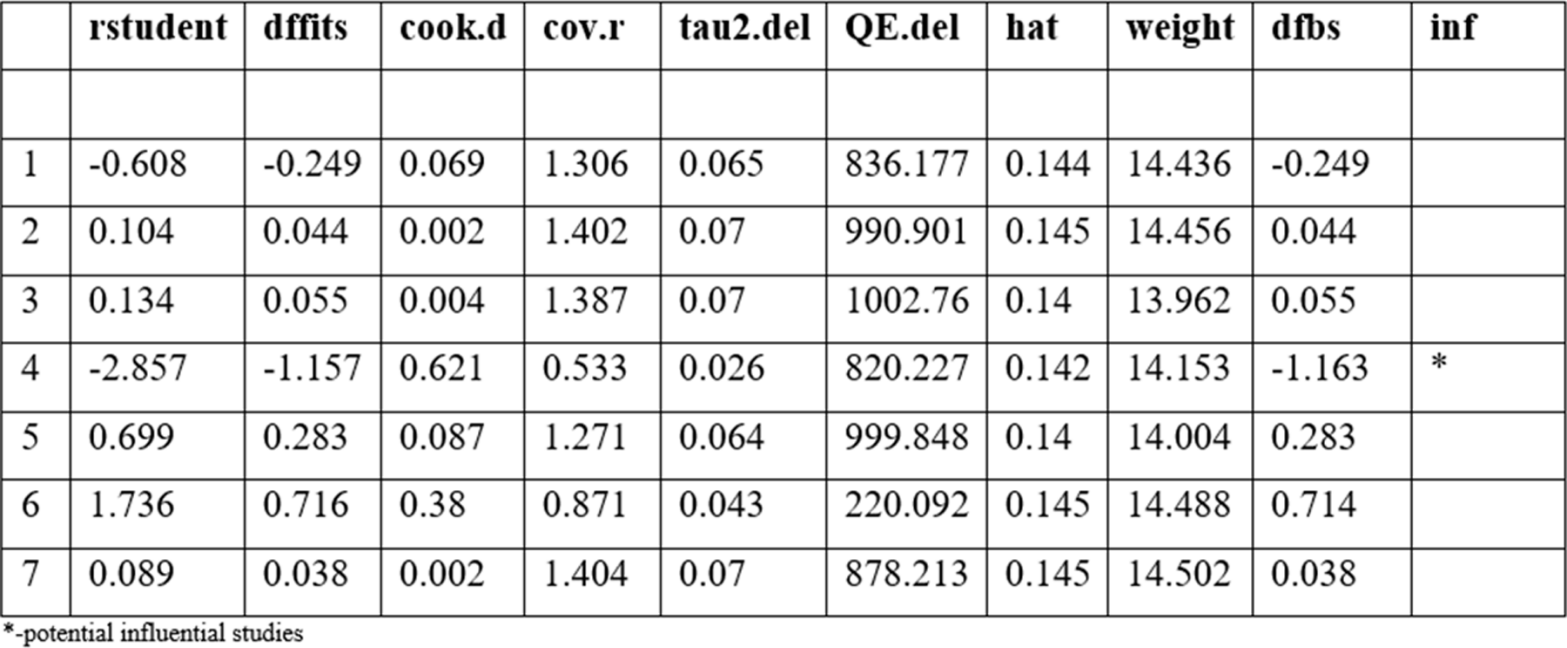
Result of the influential study test by countries.

**Fig 17:**
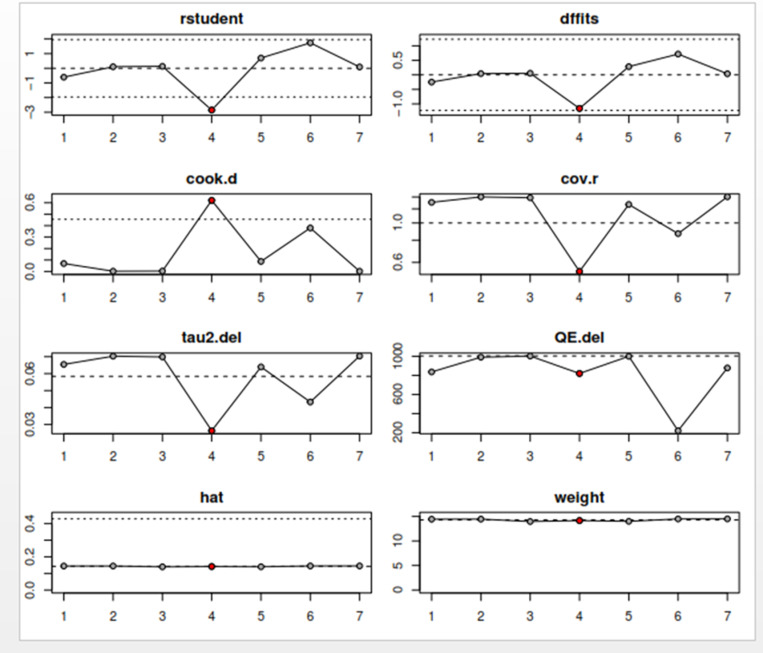
Influential study diagnostics by countries.

The Baujat plot displays extreme values (>50-contribution to overall heterogeneity) originating from the findings of three countries (Kenya, Botswana, and Zimbabwe). This suggests a potential contribution to the overall heterogeneity. In contrast, Uganda stands out as a significant outlier, exerting a considerable influence on the combined proportion (See Fig 14). Excluding Botswana significantly impacts the original summary proportion more than the latter study (See Fig 15). The leave-one-out diagnostics influential analysis suggests that Botswana is a potentially influential study. According to the Baujat plot and diagnostic test results, all four countries (Kenya, Botswana, Zimbabwe, and Uganda) could be classified as outliers, identifying Botswana as influential (See Figs 16 and 17). Hence, Botswana was removed from the pooled random estimate result (See Fig 2).

### GRADE assessment of evidence

#### Acceptability of HPV self-sampling.

The GRADE assessment revealed moderate certainty evidence supporting the high acceptability of HPV self-sampling among WLHIV. Key facilitators included privacy, convenience, and control, with clear instructions and educational support enhancing confidence.

Barriers such as concerns over improper sampling, fear of inaccuracies, and limited knowledge were common in rural and low-resource settings. The assessment shows a strong potential for implementing HPV self-sampling, particularly in urban areas with better access to resources in SSA. (S6 File)

#### Agreement between self-collected and clinician-collected samples.

The analysis revealed moderate certainty evidence supporting the agreement between self-collected and clinician-collected samples for HPV detection across diverse low-resource settings. The studies provided moderate certainty evidence for the reliability of self-collection as a viable alternative for HPV screening. While agreement metrics were generally high, limitations such as variability in genotype-specific detection and small sample sizes for certain subgroups reduce overall certainty. (S6 File)

## Discussion

This study highlights the acceptability of HPV self-sampling among WLHIV in SSA. This is the first systematic review incorporating a meta-analysis to assess the acceptability of self-sampling among WLHIV in SSA. Our findings indicate a high level of acceptability across most SSA countries included in the review, with strong agreement between self-sampling and clinician sampling results. However, acceptability rates varied, with Kenya reporting a lower rate of 49% [31,32,34]. The benefits of self-sampling, including ease of use, minimal discomfort, reduced embarrassment, and overall comfort, were key factors driving acceptability. While a previous meta-analysis by Nelson et al. reported a pooled prevalence of 59% (95% CI: 48–69) for self-sampling preference among the general female population, our study suggests potentially higher acceptability among WLHIV [44]. Similarly, findings from a scoping review reinforce the strong acceptance of HPV self-sampling in this population among WLHIV [45].

The high acceptability of self-sampling suggests that this approach could significantly improve cervical cancer screening uptake among WLHIV, particularly by offering a private, non-invasive, and convenient alternative to clinician-based sampling. This is especially crucial given the increased risk of cervical cancer among WLHIV due to their compromised immune systems [14]. However, while self-sampling was widely accepted, many WLHIV expressed concerns about its accuracy and reliability. This highlights the need for targeted interventions to enhance confidence in self-sampling methods.

To address this gap, culturally appropriate educational programs led by peers and trained healthcare professionals are essential [46]. Interactive group-based workshops using visual aids, demonstrations, and peer discussions can help dispel misconceptions, address HIV-related stigma, and provide reassurance about the validity of self-sampling. Additionally, mobile health (mHealth) platforms can be accessible tools for delivering tailored educational support, allowing WLHIV to engage with relevant resources conveniently. High-intensity educational interventions, such as those reported by Molokwu et al., have significantly improved women’s willingness to adopt self-sampling, underscoring the importance of structured educational strategies in strengthening cervical cancer prevention efforts among WLHIV [47].

Despite the strong preference for self-sampling in SSA, successful implementation requires addressing several systemic barriers. Key challenges include limited healthcare infrastructure, inadequate funding, and sociocultural barriers that may hinder access to and utilization of self-sampling services [43,44,46]. Furthermore, stigma and discrimination associated with HIV and cervical cancer screening continue to be significant obstacles. Even with a high level of acceptability, the lack of confidence among some WLHIV in self-sampling’s reliability suggests that awareness campaigns and community-based engagement strategies are necessary to ensure sustained adoption and trust in the method [48,49].

Several studies have established self-sampling as a viable alternative to clinician-collected samples for primary cervical cancer screening [8,48,49,50]. Since WHO recommends routine cervical screening and endorses self-collected samples for HPV DNA testing, self-sampling can bridge screening gaps, particularly in SSA, where screening coverage remains low [19,22,51,52]. However, despite growing evidence supporting its effectiveness, the global adoption of self-sampling remains limited [8]. As of now, only 17 countries with established cervical cancer screening programs officially endorse HPV self-sampling. In Africa, only Kenya, Rwanda, and Uganda have integrated self-sampling into their national cervical cancer screening programs [8]. This limited adoption underscores the need for stronger policy support and integration of self-sampling into routine healthcare services.

Notably, our meta-analysis confirms that self-sampling is well-accepted among WLHIV, reinforcing its potential to expand cervical cancer screening access, particularly for women in remote or underserved areas. Self-sampling aligns with participatory medicine principles, empowering women to take an active role in their health by allowing them to collect samples in the privacy and comfort of their homes [53]. This approach reduces potential discomfort, embarrassment, and logistical barriers associated with clinician-led screening [54]. Additionally, self-sampling has been identified as a cost-effective strategy for HPV screening, as it minimizes the need for clinical visits and reduces the burden on healthcare systems [55]. As a result, expanding self-sampling initiatives could improve both the affordability and accessibility of cervical cancer screening for WLHIV and other at-risk populations [55,56].

A major strength of this study is that it is the first systematic review and meta-analysis assessing the acceptability of HPV self-sampling among WLHIV in SSA. The study utilized broad search terms and multiple databases, including diverse studies across different SSA regions. Additionally, the rigorous methodological approach, including data synthesis and quality assessment, strengthens the reliability of the findings.

However, some limitations should be acknowledged. First, we excluded studies not published in English, which may have introduced selection bias. Additionally, all included studies were cross-sectional, limiting the ability to establish causal relationships. High heterogeneity in acceptability rates was observed across studies; while we excluded the most influential study to reduce bias, the small sample size prevented using meta-regression techniques to quantitatively explore the sources of heterogeneity. Furthermore, the extracted data did not consistently report associated factors in odds ratio format, limiting the ability to analyze predictors of acceptability systematically.

Further research is needed to explore interventions that enhance confidence in self-sampling among WLHIV, particularly those focused on educational strategies and community engagement. Additionally, feasibility studies assessing self-sampling integration into national cervical cancer prevention programs across SSA are essential. Future studies should also examine long-term adherence to self-sampling and its impact on cervical cancer screening rates.

## Conclusion

This study provides strong evidence supporting the acceptability of HPV self-sampling among WLHIV in SSA, with potential benefits for increasing cervical cancer screening uptake. However, challenges such as low confidence in self-sampling accuracy, healthcare infrastructure limitations, and sociocultural barriers must be addressed to maximize its impact. Strengthening educational interventions, leveraging digital health tools, and advocating for policy integration are crucial next steps in ensuring that HPV self-sampling becomes a widely accessible and trusted option for cervical cancer prevention among WLHIV in SSA.

## Supporting information

S1 File PRISMA 2020 checklist. (PDF)

S2 File Search strategy. (PDF)

S3 File Study selection. (XLSX)

S4 File Data extraction. (XLSX)

S5 File Quality assessment. (PDF)

S6 File GRADE assessment tables. (PDF)
