# Supplementary material for: Acceptability of human papillomavirus self-sampling among women living with HIV in sub-Saharan Africa: A systematic review and meta-analysis
Source: PLOS Glob Public Health. 2025 May 14;5(5):e0004605. doi: 10.1371/journal.pgph.0004605 (PMC12077793; doi:10.1371/journal.pgph.0004605)
Supplement: S2 File — (PDF) [file pgph.0004605.s002.pdf]

Search Strategy

EBSCO

#	Query	Limiters/Expanders	Last Run Via	Results
S1	TI human papillomavirus or human papilloma virus or hpv	Limiters - Full Text Expanders - Apply equivalent subjects Search modes - Find all my search terms	Interface - EBSCOhost Research Databases Search Screen - Advanced Search Database - Academic Search Ultimate;CINAHL Complete;Business Source Ultimate;OpenDissertations;eBook Collection (EBSCOhost);EBSCO eClassics Collection (EBSCOhost);ERIC;Newspaper Source;European Views of the Americas: 1493 to 1750;GreenFILE;Library, Information Science & Technology Abstracts;MEDLINE;Newswires;Regional Business News;Teacher Reference Center;APA PsycTherapy	20,211
S2	TI acceptance OR TI acceptability	Limiters - Full Text Expanders - Apply equivalent subjects Search modes - Find all my search terms	Interface - EBSCOhost Research Databases Search Screen - Advanced Search Database - Academic Search Ultimate;CINAHL Complete;Business Source Ultimate;OpenDissertations;eBook Collection (EBSCOhost);EBSCO eClassics Collection (EBSCOhost);ERIC;Newspaper Source;European Views of the Americas: 1493 to 1750;GreenFILE;Library, Information Science & Technology Abstracts;MEDLINE;Newswires;Regional Business News;Teacher Reference Center;APA PsycTherapy	31,061
S3	TI human immunodeficiency syndrome OR HIV	Limiters - Full Text Expanders - Apply equivalent subjects Search modes - Find all my search terms	Interface - EBSCOhost Research Databases Search Screen - Advanced Search Database - Academic Search Ultimate;CINAHL Complete;Business Source Ultimate;OpenDissertations;eBook Collection (EBSCOhost);EBSCO eClassics Collection (EBSCOhost);ERIC;Newspaper Source;European Views of the Americas: 1493 to 1750;GreenFILE;Library, Information Science & Technology Abstracts;MEDLINE;Newswires;Regional Business News;Teacher Reference Center;APA PsycTherapy	287,553
S4	self test or self sampling	Limiters - Full Text Expanders - Apply equivalent subjects Search modes - Find all my search terms	Interface - EBSCOhost Research Databases Search Screen - Advanced Search Database - Academic Search Ultimate;CINAHL Complete;Business Source Ultimate;OpenDissertations;eBook Collection (EBSCOhost);EBSCO eClassics Collection (EBSCOhost);ERIC;Newspaper	201,914

			Source;European Views of the Americas: 1493 to 1750;GreenFILE;Library, Information Science & Technology Abstracts;MEDLINE;Newswires;Regional Business News;Teacher Reference Center;APA PsycTherapy	
S5	S1 AND S2 AND S3 AND S4	Limiters - Full Text<br>Expanders - Apply equivalent subjects<br>Search modes - Find all my search terms	Interface - EBSCOhost Research Databases<br>Search Screen - Advanced Search<br>Database - Academic Search Ultimate;CINAHL Complete;Business Source Ultimate;OpenDissertations;eBook Collection (EBSCOhost);EBSCO eClassics Collection (EBSCOhost);ERIC;Newspaper Source;European Views of the Americas: 1493 to 1750;GreenFILE;Library, Information Science & Technology Abstracts;MEDLINE;Newswires;Regional Business News;Teacher Reference Center;APA PsycTherapy	8

Academic Search Ultimate (5)
CINAHL 3

Web of Science - 17

•	Query	Results
#1	**((TS=(ACCEPTANCE)) OR TS=(ACCEPTABILITY)) NOT (SILOID==("PPRN"))**	357,538
#2	**(((TS=("SELF-SAMPLING")) OR TS=("self-testing" or "self_test")) OR TS=("Self-collected sample")) NOT (SILOID==("PPRN"))**	8,193
#3	**(TS=(human papillomavirus or human papilloma virus or hpv)) NOT (SILOID==("PPRN"))**	107,362
#4	**((((TS=(hiv or aids or acquired human immunodeficiency syndrome or human immunodeficiency virus)) OR TS=(hiv or aids or acquired human immunodeficiency syndrome or human immunodeficiency virus)) OR TS=(HIV)) OR TS=(WLHIV)) NOT (SILOID==("PPRN"))**	1,550,795
#5	**#1 AND #2 AND #3 AND #4** and **Preprint Citation Index** (Exclude – Database)	30
#6	**#1 AND #2 AND #3 AND #4** and **Preprint Citation Index** (Exclude – Database) and **CAMEROON** or **BOTSWANA** or **ETHIOPIA** or **GHANA** or **TANZANIA** or **KENYA** or **MALAWI** or **MALI** or **NIGERIA** or **SENEGAL** or **SOUTH AFRICA** or **COTE IVOIRE** (Countries/Regions)	17

PUBMED- 9

	Query	Results
#1	"SELF-SAMPLING"[Title/Abstract] OR "self collected sample"[Title/Abstract] OR "Self-examination"[Title/Abstract] OR "Self-obtained"[Title/Abstract] OR "Self-assessment"[Title/Abstract]	19,348
#2	"ACCEPTABILITY"[Title/Abstract] OR "ACCEPTANCE"[Title/Abstract]	144,281

	"human papillomavirus viruses"[MeSH Terms] OR "HPV"[Title/Abstract	54,337
	"HIV"[MeSH Terms] OR "women living with hiv"[Title/Abstract] OR "HIV"[Title/Abstract]	372,568
#3	("HIV"[MeSH Terms] OR "women living with hiv"[Title/Abstract] OR "HIV"[Title/Abstract]) AND ("human papillomavirus viruses"[MeSH Terms] OR "HPV"[Title/Abstract]) AND ("ACCEPTABILITY"[Title/Abstract] OR "ACCEPTANCE"[Title/Abstract]) AND ("SELF-SAMPLING"[Title/Abstract] OR "self collected sample"[Title/Abstract] OR "Self-examination"[Title/Abstract] OR "Self-obtained"[Title/Abstract] OR "Self-assessment"[Title/Abstract])	18
#4	("BENIN"[Title/Abstract] OR "BOTSWANA"[Title/Abstract] OR "burkina faso"[Title/Abstract] OR "BURUNDI"[Title/Abstract] OR "cabo verde"[Title/Abstract] OR "CAMEROON"[Title/Abstract] OR "central african republic"[Title/Abstract] OR "CHAD"[Title/Abstract] OR "COMOROS"[Title/Abstract] OR (("congo"[MeSH Terms] OR "congo"[All Fields]) AND "dem rep"[Title/Abstract]) OR (("congo"[MeSH Terms] OR "congo"[All Fields]) AND "rep"[Title/Abstract]) OR "cote d ivoire"[Title/Abstract] OR "equatorial guinea"[Title/Abstract] OR "ERITREA"[Title/Abstract] OR "ESWATINI"[Title/Abstract] OR "ETHIOPIA"[Title/Abstract] OR "GABON"[Title/Abstract] OR "GAMBIA"[Title/Abstract] OR "GHANA"[Title/Abstract] OR "GUINEA"[Title/Abstract] OR "GUINEA-BISSAU"[Title/Abstract] OR "KENYA"[Title/Abstract] OR "LESOTHO"[Title/Abstract] OR "LIBERIA"[Title/Abstract] OR "MADAGASCAR"[Title/Abstract] OR "MALAWI"[Title/Abstract] OR "MALI"[Title/Abstract] OR "MAURITANIA"[Title/Abstract] OR "MAURITIUS"[Title/Abstract] OR "MOZAMBIQUE"[Title/Abstract] OR "NAMIBIA"[Title/Abstract] OR "NIGER"[Title/Abstract] OR "NIGERIA"[Title/Abstract] OR "RWANDA"[Title/Abstract] OR ("sao tome"[Title/Abstract] AND "PRINCIPE"[Title/Abstract]) OR "SENEGAL"[Title/Abstract] OR "SEYCHELLES"[Title/Abstract] OR "sierra leone"[Title/Abstract] OR "SOMALIA"[Title/Abstract] OR "south africa"[Title/Abstract] OR "south sudan"[Title/Abstract] OR "SUDAN"[Title/Abstract] OR "TANZANIA"[Title/Abstract] OR "TOGO"[Title/Abstract] OR "UGANDA"[Title/Abstract] OR "ZAMBIA"[Title/Abstract] OR "ZIMBABWE"[Title/Abstract]) AND 1990/01/01:2022/12/31[Date - Publication]	283,968
	("HIV"[MeSH Terms] OR "women living with hiv"[Title/Abstract] OR "HIV"[Title/Abstract]) AND ("human papillomavirus viruses"[MeSH Terms] OR "HPV"[Title/Abstract]) AND ("ACCEPTABILITY"[Title/Abstract] OR "ACCEPTANCE"[Title/Abstract]) AND ("SELF-SAMPLING"[Title/Abstract] OR "self collected sample"[Title/Abstract] OR "Self-examination"[Title/Abstract] OR "Self-obtained"[Title/Abstract] OR "Self-assessment"[Title/Abstract]) AND (("BENIN"[Title/Abstract] OR "BOTSWANA"[Title/Abstract] OR "burkina faso"[Title/Abstract] OR "BURUNDI"[Title/Abstract] OR "cabo verde"[Title/Abstract] OR "CAMEROON"[Title/Abstract] OR	9

	"central african republic"[Title/Abstract] OR "CHAD"[Title/Abstract] OR "COMOROS"[Title/Abstract] OR (("congo"[MeSH Terms] OR "congo"[All Fields]) AND "dem rep"[Title/Abstract]) OR (("congo"[MeSH Terms] OR "congo"[All Fields]) AND "rep"[Title/Abstract]) OR "cote d ivoire"[Title/Abstract] OR "equatorial guinea"[Title/Abstract] OR "ERITREA"[Title/Abstract] OR "ESWATINI"[Title/Abstract] OR "ETHIOPIA"[Title/Abstract] OR "GABON"[Title/Abstract] OR "GAMBIA"[Title/Abstract] OR "GHANA"[Title/Abstract] OR "GUINEA"[Title/Abstract] OR "GUINEA-BISSAU"[Title/Abstract] OR "KENYA"[Title/Abstract] OR "LESOTHO"[Title/Abstract] OR "LIBERIA"[Title/Abstract] OR "MADAGASCAR"[Title/Abstract] OR "MALAWI"[Title/Abstract] OR "MALI"[Title/Abstract] OR "MAURITANIA"[Title/Abstract] OR "MAURITIUS"[Title/Abstract] OR "MOZAMBIQUE"[Title/Abstract] OR "NAMIBIA"[Title/Abstract] OR "NIGER"[Title/Abstract] OR "NIGERIA"[Title/Abstract] OR "RWANDA"[Title/Abstract] OR ("sao tome"[Title/Abstract] AND "PRINCIPE"[Title/Abstract]) OR "SENEGAL"[Title/Abstract] OR "SEYCHELLES"[Title/Abstract] OR "sierra leone"[Title/Abstract] OR "SOMALIA"[Title/Abstract] OR "south africa"[Title/Abstract] OR "south sudan"[Title/Abstract] OR "SUDAN"[Title/Abstract] OR "TANZANIA"[Title/Abstract] OR "TOGO"[Title/Abstract] OR "UGANDA"[Title/Abstract] OR "ZAMBIA"[Title/Abstract] OR "ZIMBABWE"[Title/Abstract]) AND 1990/01/01:2022/12/31[Date - Publication])	

COCHRANE -8

#1 human papillomavirus 2910
#2 HPV 3874
#3 #1 OR #2 4324
#4 "SELF SAMPLING" 294
#5 "SELF TESTING" 616
#6 "Self-obtained" 22
#7 Self-examination 694
#8 #4 OR #5 OR #6 OR #7 1579
#9 ACCEPTABILITY 23210
#10 ACCEPTANCE 17084
#11 #9 OR #10 38144
#12 Human Immunodeficiency Virus 14469
#13 HIV 32998
#14 WLHIV 18
#15 #12 OR #13 OR #14 33755
#16 #3 AND #8 AND #11 AND #15 8

GOOGLE SCHOLAR

998
