# Supplementary material for: Acceptability of human papillomavirus self-sampling among women living with HIV in sub-Saharan Africa: A systematic review and meta-analysis
Source: PLOS Glob Public Health. 2025 May 14;5(5):e0004605. doi: 10.1371/journal.pgph.0004605 (PMC12077793; doi:10.1371/journal.pgph.0004605)
Supplement: S5 File — (PDF) [file pgph.0004605.s005.pdf]

Supplementary File 2

Quality Assessment_ HPV Self-Sampling

Quality assessment for included studies using Appraisal Tool for Cross-Sectional Studies (AXIS)

	**Introduction**	**Methods**									
**Author (Year of publication)**	**Were the aims/objectives of the study clear?**	**Was the study design appropriate for the stated aim(s)?**	**Was the sample size justified?**	**Was the target/ reference population clearly defined? (Is it clear who the research was about?)**	**Was the sample frame taken from an appropriate population base so that it closely represented the target/reference population under investigation?**	**Was the selection process likely to select subjects/participants that were representative of the target/ reference population under investigation?**	**Were measures undertaken to address and categorize non-responders?**	**Were the risk factors and outcome variables measured appropriate to the aims of the study?**	**Were the risk factors and outcome variables measured correctly using instruments/ measurements that had been trialed, piloted or published previously?**	**Is it clear what was used to determined statistical significance and/or precision estimates? (e.g., p values,CIs)**	**Were the methods (including statistical methods) sufficiently described to enable them to be repeated?**
Adamson *et al.*, (2015)	Y	Y	Y	Y	Y	Y	N	Y	CT	Y	Y
Rositch *et al.*, (2012)	Y	Y	N	Y	Y	Y	N	Y	CT	Y	Y
Grabert *et al.*,(2022)	Y	Y	N	Y	Y	Y	N	Y	N	N	N
Kohler *et al.*, (2019)	Y	Y	N	Y	Y	Y	N	Y	CT	Y	Y
Islam *et al.*, (2020)	Y	Y	N	Y	Y	Y	N	Y	N	Y	Y
Sormani *et al.*, (2021)	Y	Y	N	Y	Y	Y	N	Y	N	Y	Y
Bansil *et al.*, (2014)	Y	Y	N	Y	Y	Y	N	Y	N	Y	Y
Taku *et al.*, (2020)	Y	Y	N	Y	Y	Y	N	Y	N	N	N
Joseph *et al.*, (2021)	Y	Y	N	Y	Y	Y	N	Y	N	Y	Y
Mahomed *et al.*, (2014)	Y	Y	N	Y	Y	Y	N	Y	Y	Y	Y
Obiri-Yeboah *et al.*, (2017)	Y	Y	N	Y	Y	Y	N	Y	CT	Y	Y
Mitchell *et al.*, (2017)	Y	Y	N	Y	Y	Y	N	Y	Y	Y	Y
Mbatha *et al.*, (2017)	y	y	N	y	y	y	N	y	CT	Y	Y
Nyabigambo *et al.*, (2022)	y	y	Y	y	y	y	N	y	CT	Y	Y

Note: Y = Yes, N = No, CT= Cannot Tell

Quality assessment for included studies using Appraisal Tool for Cross-Sectional Studies (AXIS)

	Result					Discussion		Others		
**Author (Year of publication)**	**Were the basic data adequately described?**	**Does the response rate raise concerns about non-response bias?**	**If appropriate, was information about non-responders described?**	**Were the results internally consistent?**	**Were the results for the analyses described in the methods presented?**	**Were the authors' discussions and conclusions justified by the results?**	**Were the limitations of the study discussed?**	**Were there any funding sources or conflicts of interest that may affect the authors' interpretation of the results?**	**Was ethical approval or consent of participants attained?**	**Overall score**
Adamson *et al.*, (2015)	Y	N	N	Y	Y	Y	Y	N	Y	17
Rositch *et al.*, (2012)	Y	N	N	Y	Y	Y	Y	N	Y	16
Grabert *et al.*,(2022)	Y	N	N	Y	Y	Y	Y	N	Y	14
Kohler *et al.*, (2019)	Y	N	N	Y	Y	Y	Y	N	Y	16
Islam *et al.*, (2020)	Y	N	N	Y	N	Y	Y	N	Y	15
Sormani *et al.*, (2021)	Y	N	N	Y	Y	Y	Y	N	Y	16
Bansil *et al.*, (2014)	N	N	N	Y	Y	Y	Y	N	Y	15
Taku *et al.*, (2020)	Y	N	N	Y	Y	Y	Y	N	Y	14
Joseph *et al.*, (2021)	N	N	N	Y	Y	Y	Y	N	Y	15
Mahomed *et al.*, (2014)	Y	N	N	Y	Y	Y	Y	Y	Y	18
Obiri-Yeboah *et al.*, (2017)	Y	N	N	Y	Y	Y	Y	N	Y	16
Mitchell *et al.*, (2017)	N	N	N	Y	Y	Y	Y	N	Y	16
Mbatha *et al.*, (2017)	Y	N	N	Y	Y	Y	Y	N	Y	16
Nyabigambo *et al.*, (2022)	N	N	N	Y	N	Y	Y	N	Y	15

Note: Y = Yes, N = No, CT= Cannot Tell
