# Supplementary material for: Acceptability of human papillomavirus self-sampling among women living with HIV in sub-Saharan Africa: A systematic review and meta-analysis
Source: PLOS Glob Public Health. 2025 May 14;5(5):e0004605. doi: 10.1371/journal.pgph.0004605 (PMC12077793; doi:10.1371/journal.pgph.0004605)
Supplement: S6 File — (PDF) [file pgph.0004605.s006.pdf]

Supplementary File 3
GRADE Assessment of Acceptability of Self sampling

S/No	Article	Risk of Bias	Inconsistency	Indirectness	Imprecision	Publication Bias	Overall Certainty
1.	Adamson *et al.*, (2015)	Low	Moderate	Low	Moderate	Low	Moderate
2.	Rositch *et al.*, (2012)	Moderate	Low	Moderate	Moderate	Low	Moderate
3.	Taku *et al.*, (2020)	Moderate	Low	Moderate	Moderate	Low	Moderate
4.	Joseph et al., 2021	Moderate	Low	Moderate	Moderate	Low	Moderate
5.	Kohler et al., 2019	Low	Low	Moderate	Moderate	Low	Moderate
6.	Mahomed et al., 2014	Moderate	Low	Moderate	Moderate	Low	Moderate
7.	Obiri-Yeboah et al., 2017	Low	Low	Moderate	Moderate	Low	Moderate
8.	Mitchell et al., 2017	Moderate	Low	Moderate	Moderate	Low	Moderate
9.	Mbatha et al., 2017	Moderate	Low	Moderate	Moderate	Low	Moderate
10.	Nyabigambo et al., 2022	Moderate	Low	Moderate	Moderate	Low	Moderate
11.	Grabert et al., 2022	Moderate	Low	Moderate	Moderate	Low	Moderate
12.	Islam et al., 2021	Low	Low	Moderate	Moderate	Low	Moderate
13.	Bansil et al., 2014	Moderate	Low	Moderate	Moderate	Low	Moderate
14.	Sormani et al., 2021	Moderate	Low	Moderate	Moderate	Low	Moderate

Table 3: GRADE Assessment of Agreement

S/No	Article	Risk of Bias	Inconsistency	Indirectness	Imprecision	Publication Bias	Overall Certainty
1.	Bansil et al., (2014)	Moderate	Low	Moderate	Moderate	Low	Moderate
2.	Joseph et al., (2021)	Moderate	Low	Moderate	Moderate	Low	Moderate
3.	Obiri-Yeboah et al., (2017)	Moderate	Low	Moderate	Moderate	Low	Moderate
4.	Taku et al., (2020)	Moderate	Low	Moderate	Moderate	Low	Moderate

Agreement Between Self-Collected and Clinician-Collected Samples

The analysis revealed **moderate certainty evidence** supporting the agreement between self-collected and clinician-collected samples for HPV detection across diverse low-resource settings. The studies provided **moderate certainty evidence** for the reliability of self-collection as a viable alternative for HPV screening. While agreement metrics were generally high, limitations such as variability in genotype-specific detection and small sample sizes for certain subgroups reduce overall certainty.
